# Vascular injuries after blunt chest trauma: diagnosis and management

**DOI:** 10.1186/1757-7241-17-42

**Published:** 2009-09-14

**Authors:** James V O'Connor, Christopher Byrne, Thomas M Scalea, Bartley P Griffith, David G Neschis

**Affiliations:** 1 Program in Ttauma, R. Adams Cowley Shock Trauma Center, Baltimore, USA; 2 Department of Surgery, University of Maryland School of Medicine, Baltimore, USA

## Abstract

**Background:**

Although relatively rare, blunt injury to thoracic great vessels is the second most common cause of trauma related death after head injury. Over the last twenty years, the paradigm for management of these devastating injuries has changed drastically. The goal of this review is to update the reader on current concepts of diagnosis and management of blunt thoracic vascular trauma.

**Methods:**

A review of the medical literature was performed to obtain articles pertaining to both blunt injuries of the thoracic aorta and of the non-aortic great vessels in the chest. Articles were chosen based on authors' preference and clinical expertise.

**Discussion:**

Blunt thoracic vascular injury remains highly lethal, with most victims dying prior to reaching a hospital. Those arriving in extremis require immediate intervention, which may include treatment of other associated life threatening injuries. More stable injuries can often be medically temporized in order to optimize definitive management. Endovascular techniques are being employed with increasing frequency and can often significantly simplify management in otherwise very complex patient scenarios.

## Introduction

Blunt thoracic great vessel trauma is relatively rare; representing less than 5% of traumatic vascular injuries, with penetrating mechanism predominating [1]. The true incidence is likely underestimated, as many victims die prior to arriving at the hospital for definitive treatment[2]. Of those alive on hospital admission, traumatic aortic rupture accounts for the vast majority of blunt thoracic vascular injuries [3]. With an estimated incidence of 7,500 - 8,000 cases per year in the United States, blunt thoracic aortic trauma is the second most common cause of trauma related death after head injury[4]. Most traumatic aortic injuries are fatal at the scene of the accident in up to 80-90% of cases [5]. Thoracic aortic rupture accounts for nearly 18% of all deaths in motor vehicle collisions[6]. Patients often sustain injuries to multiple organ systems including head, pulmonary, and abdominal injury. Regardless of location, however, blunt injuries to the thoracic vasculature are highly lethal injuries requiring timely diagnosis and life saving intervention. This review will focus on diagnosis and management of blunt injuries of both the non-aortic thoracic vessels as well as blunt injury of the thoracic aorta.

The number of patients with blunt thoracic vascular injuries, not including those with traumatic aortic rupture, is quite small. The analysis of this patient subset is further hampered by the fact that most of the published reports consists of case series [7-11], studies combining both blunt and penetrating trauma [12-15], and those combining subclavian and axillary injuries [16-18]. There are few large series limited to blunt injury of the thoracic great vessels [19,20].

In addition to thoracic aortic injury, the thoracic great vessels to be discussed are the innominate artery and veins, subclavian artery and veins, left common carotid artery, pulmonary artery and veins, azygous vein, intra-thoracic vena cavae, and combined airway and vascular trauma. The innominate artery accounts for approximately half of the injuries with subclavian and left common carotid arteries accounting for almost all the remainder [19]. Pulmonary vessels, azygous vein, and caval injuries are quite rare.

Comparing those patients with penetrating versus blunt thoracic great vessel injury is illustrative. In general, penetrating injuries result in higher mortality, more combined arterial and venous injures, and lower morbidity than those presenting with blunt trauma [12-14,17]. Mortality for blunt injury has been reported between zero and 24%[8,12,14,19]. Associated extra-thoracic injures, especially abdominal and cerebral, are common and may contribute to mortality [2,12,13,15]. Morbidity, including amputation and brachial plexus injury, is frequent, and may result in long term disability[8,9,17,19,21].

### Mechanism of Injury

Descending thoracic aortic injuries are associated with high speed motor vehicle collisions (>60 miles per hour) (100 km/hr), high injury severity scores, and often coupled with significant associated injuries. A prospective study of blunt aortic injury admissions showed that most occurred after head on collisions (72%), while side impact (24%) and rear impact (4%) collisions accounted less often [22]. Blunt thoracic aortic injury is strongly correlated with a change in velocity of 20 mph (32 km/h) or more, near-side impact, and significant vehicle damage with intrusion of the wall into the passenger compartment of 15 inches (40 cm) or more, and is not correlated with use of seat belts or airbags [23]. The overall incidence of blunt aortic injury has remained the same over the past 12 years despite advances in vehicle restraint systems [24].

Blunt aortic injury is thought to occur after sudden deceleration and tearing of the aorta at the transition from mobile to fixed thoracic aorta, usually at the aortic isthmus distal to the origin of the left subclavian artery (ligamentum arteriosum). A landmark study by Parmley described 45% of the blunt thoracic aortic injuries occurred at this location [5]. Shear forces and stretching of the aorta are likely mechanisms of injury. "Pinch injury" as an alternative or additional cause has also been suggested[25]. In this scenario the aortic isthmus is violently compressed by the first rib. A theoretical sequence of injury involves rupture of the inner intimal and medial layers with subsequent delayed rupture of the adventitia. This window prior to complete rupture is the rationale for timely diagnosis and treatment.

Similar mechanisms are implicated in the injury of the non-aortic great vessels as well. Hyperextension and traction on blood vessels have been postulated as additional mechanisms[7]. Regardless of the mechanism or mechanisms, the result is vessel wall disruption, occlusion, or avulsion. Shearing can result in all of these and compression more often results in occlusion. A small intimal disruption can lead to thrombus formation and occlusion. If the mechanism of injury results in vessel avulsion the patient may die prior to arriving at the hospital[2], or may not survive operation [19,26]. More commonly, thoracic trauma results in arterial wall disruption with pseudoaneurysm formation, which may not become symptomatic until years later [27].

Innominate artery and left carotid injuries almost always occur proximally at the vessel origin[19,20,28]. In contrast, blunt subclavian injuries tend to be more distal[8,12]. While several theories have been postulated, the exact mechanism remains unknown.

### Evaluation and Imaging

The history may be obtained from the patient but more likely will be provided to the medical staff by pre-hospital personnel. If the patient was involved in a motor vehicle collision, information about restraint use, airbag deployment, occupant compartment intrusion, and injures or death of other vehicle occupants can provide clues to crash severity. If the mechanism was a fall from height, the distance the victim fell, the surface struck, and position on landing may yield valuable information.

The clinical picture of patients with blunt great vessel injury varies from asymptomatic to profound shock. On inspection, signs of chest wall trauma may be absent. In one series admission hypotension was common [19], while in others it occurred infrequently but was an ominous finding [20]. The physical findings related to arterial occlusion include an absent or diminished upper extremity pulse and differential upper extremity blood pressures. The presence of a palpable pulse does not exclude an arterial injury since there is excellent collateral flow around the shoulder. Although uncommon, the presence of a thrill or bruit should alert the physician to the presence of a vascular injury. A thorough neurologic examination is essential as it may help guide therapy and predict long term limb function. Specific evaluation of the brachial plexus is mandatory as there is a strong correlation between a brachial plexopathy and thoracic vascular injuries, especially the subclavian artery[21,29]. Additionally, hemispheric neurologic findings may alert the clinician to injury of the innominate or carotid arteries. Associated injures are common, need to be fully evaluated, and may impact survival[8,9,19]. A portable chest radiograph provides essential information as it may demonstrate a pneumothorax, hemothorax, rib fractures, or a widened mediastinum. Mediastinal widening is the most common radiographic finding with blunt great vessel injury and warrants further investigation [8,19,20]. Unlike the evaluation for a descending thoracic aortic rupture there is only a minimal role for transesophageal echocardiography in the assessment of blunt great vessel injury. Similarly, while color flow Doppler has been advocated it has not been widely employed [16].

Blunt aortic injury should be considered when mechanism is appropriate (fall, high speed MVC, pedestrian struck by auto). Symptoms include interscapular pain, dyspnea, dysphagia, signs of chest wall trauma (steering wheel imprint), new cardiac or interscapular murmur, left supraclavicular hematoma, or relative upper extremity hypertension ("pseudo-coarctation"). Signs of aortic rupture on plain radiography include mediastinal widening (>8 cm), loss of aortico-pulmonary window, tracheal deviation to the right, nasogastric shifting to right, left apical cap, depression of the left mainstem bronchus, left sided pleural effusions, or scapular, sternal, thoracic spine or multiple rib fractures[30].

**Figure 1:**
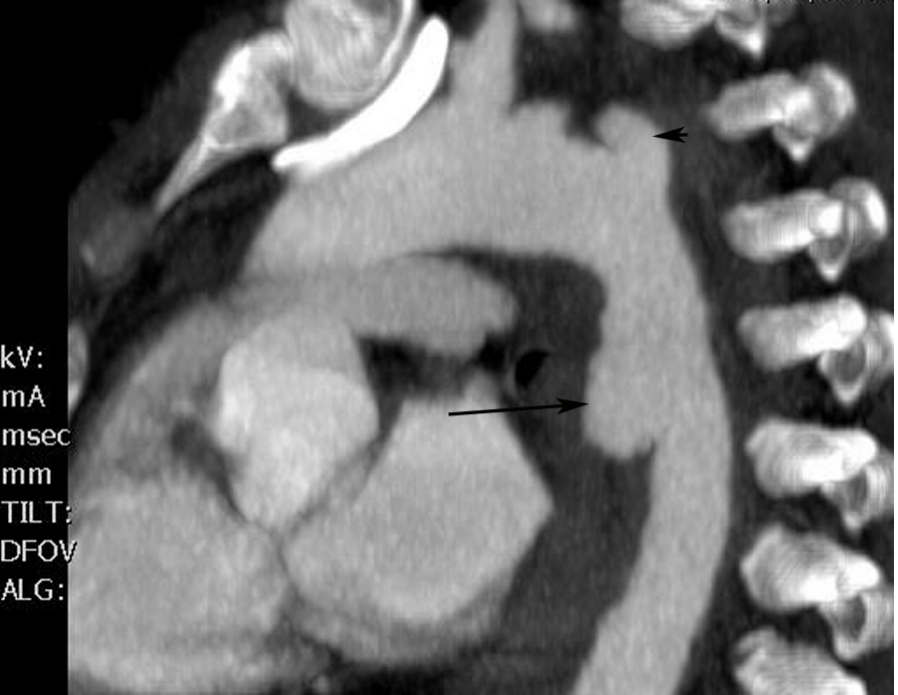
**Reconstructed computed tomography with contrast depicting aortic injury with pseudoaneurysm (arrow)**. Arrowhead indicates proximal left subclavian artery.

Historically bi-planar angiography has been the diagnostic modality of choice for evaluating blunt great vessel and aortic injury based on the landmark study by Parmley [5]. However, aortography is invasive and requires a special team for its performance and is therefore not a good screening study. In the past, the risk of a missed injury in these cases had been considered too great by some and routine screening by aortography had been suggested[4]. This dilemma is now largely only of historical interest since the advent of modern computed tomagraphy (CT) technology[31,32]. CT has sensitivities of 97-99.3% and specificities of 87.1-99.8% and routine use before angiography resulted in cost savings of greater than $365,000 over a four year period[31]. CT is now the diagnostic test of choice (Figure 1) [31,33]. The same can not be said for the use of CT scanning for the diagnosis of blunt injury to aortic branch vessels. The small number of patients undergoing CT for aortic branch vessel trauma and questions as to its accuracy has limited its use as the diagnostic test of choice [34,35]. New generation, multiple detector CT technology, however, has clearly improved diagnostic quality and reduced the need for catheter based angiography. Our practice is similar to others as we use CT as a screening test and angiography as needed [35,36]. Magnetic resonance imaging, transesophageal echocardiography, and intravascular ultrasonography are alternative modalities in particular for diagnosis of blunt aortic injury.

### Initial Management

The initial management of patients with suspected blunt great vessel injury is similar to that of any trauma patient. While a comprehensive discussion of the assessment of the trauma patient is beyond the scope of this article, a few salient points require mentioning. The primary survey of airway, breathing, circulation, disability and exposure (ABCDE) with concomitant treatment of life-threatening injuries remains the cornerstone in evaluating these patients. A more detailed examination during the secondary survey, chest and pelvic plain radiographs, and the use of Focused Assessment with Sonography for Trauma (FAST) allows the formulation of an initial plan. The overall plan depends on the clinical situation, constellation of injuries, and hemodynamic stability. Treatment may be immediate operation, further imaging studies, or expectant/non-operative management. Clinical judgment is paramount and life threatening injuries take precedence. While associated injuries are common, often the great vessel injury takes priority[8,9,19,20]. The need for emergent surgery is based on hemodynamics; as the unstable hypotensive patient may need rapid control of hemorrhage [19,20]. In particular for aortic injuries, timing of repair is based both on the extent of the patient's coexisting injuries as well as the extent of injury to the thoracic aorta. Small pseudoaneurysms and intimal injuries that don't appear to penetrate the outer wall of the aorta can generally be managed expectantly, reserving treatment for lesions that do not spontaneously resolve. Lesions with evidence of significant mediastinal hematoma need to be managed more aggressively. It should be noted however, that there is evidence that as many as 50% of minimal injury lesions (defined as an intimal flap of less than 1 cm with no or minimal periaortic hematoma) can develop into pseuoaneuysms at 8 week follow-up [37]. It is likely that the less invasive nature of endograft repair will allow more options for patients who were previously managed non-operatively.

The initial management of hemodynamically stable patients may include the use of β-blockers to lower the mean arterial pressure and to decrease aortic shear force (dP/dt). The target mean arterial pressure is between 60 and 70 mmHg. This approach has been extrapolated from the initial treatment of traumatic aortic rupture for which a delayed approach is being employed with increasing frequency[28,38]. A prospective study used beta-blockers with or without vasodilators to keep systolic blood pressures near 100 mm Hg, and a heart rate below 100 beats per minute in selected patients with blunt aortic injury and either concomitant head injury, pulmonary injury or cardiac insufficiency. There were no treatment failures prior to delayed aortic repair[39].

However, if there is a significant associated cerebral injury, even mild hypotension may worsen the neurologic outcome and normal blood pressure should be maintained. The data on hypotensive resuscitation is mixed, and a good review of this interesting topic is available[40]. In a randomized study of 598 patients with penetrating trauma, there was a significant survival benefit in the group which did not receive fluid, especially among those with cardiac injury[41]. This study has several important limitations; it is limited to penetrating injury and cardiac injuries represent a special subset of patients where survival may be more a function of time to surgery and the presence of tamponade. A randomized study from our institution showed no difference in mortality between those patients treated with normotensive versus hypotensive fluid resuscitation[42].

The concept of damage control surgery for penetrating abdominal trauma was introduced in 1993 and has expanded to other cavitary injuries[43,44]. If shock, coagulapathy, and hypothermia are not arrested, death will ensue. These same principles can be applied to vascular and thoracic trauma. With regard to vascular surgery, temporary arterial shunts allow distal perfusion and delayed vascular reconstruction. They are easy to insert and have an excellent patency rate, especially for proximal extremity vessels[45,46]. Another technique is the use of prosthetic grafts, even in contaminated wounds, as a temporizing maneuver prior to revascularization with autogenous conduit [47]. The principles of thoracic damage control are not as straight forward. In addition to hemorrhage, hypoxia and hypercarbia can also be lethal. The surgical approach consists of an abbreviated operation using non-anatomic pulmonary resection and temporary chest closure with delayed definitive closure[48,49].

### Definitive Treatment

Definitive treatment can be divided into operative procedures and the placement of endoluminal stent grafts. Some general principles will be discussed followed by the treatment of specific vessel injury. Several incisions have been used to obtain exposure of the great vessels. There is agreement that median sternotomy, with clavicular or neck extension if needed, is it the preferred approach for the majority of great vessel trauma, including the right subclavian artery. There is still some debate as to optimal exposure of the proximal left subclavian artery with some advocating a high antero-lateral thoracotomy combined with a clavicular incision[3,50]. Others, our group included, prefer to approach the proximal left subclavian using a sternotomy with extension if needed, as it provides excellent exposure[51,52]. Division of the innominate vein will greatly improve exposure. Regardless of the operative approach, intra-operative blood salvage, large bore intravenous access, and communication with the anesthesia team are essential.

While there are various techniques to manage vessel injury it is imperative to adhere to the general principles and techniques of vascular surgery. Given the arterial diameter of the great vessels, most will require prosthetic graft interposition and less commonly the injury is amenable to autogolous vein or primary repair. Ligation of the subclavian artery should be considered as a life-saving procedure in the moribund patient. With the exception of the cavae, most large veins can be ligated. In stable patients lateral venorrophy should be employed if it does not result in stenosis.

While there has been a substantial increase in the number of traumatic aortic ruptures treated with endovascular intervention, this technique has limited utility in the treatment of aortic branch vessel injury. There are several factors which limit the use of endovascular techniques to aortic branch vessels. As with traumatic aortic rupture, hemodynamic unstable patients will undergo surgery thus limiting transcather therapy to those who are hemodynamically stable. With the exception of the subclavian artery, most great vessel injuries are proximal at the origin of the artery from the aortic arch[19,20,28]. This anatomic location often precludes the use of the adjacent vessel as a landing zone since it does not have adequate length and, it may not be possible to preserve adjacent vessels[53,54].

### Specific Injuries

#### Innominate artery

**Figure 2:**
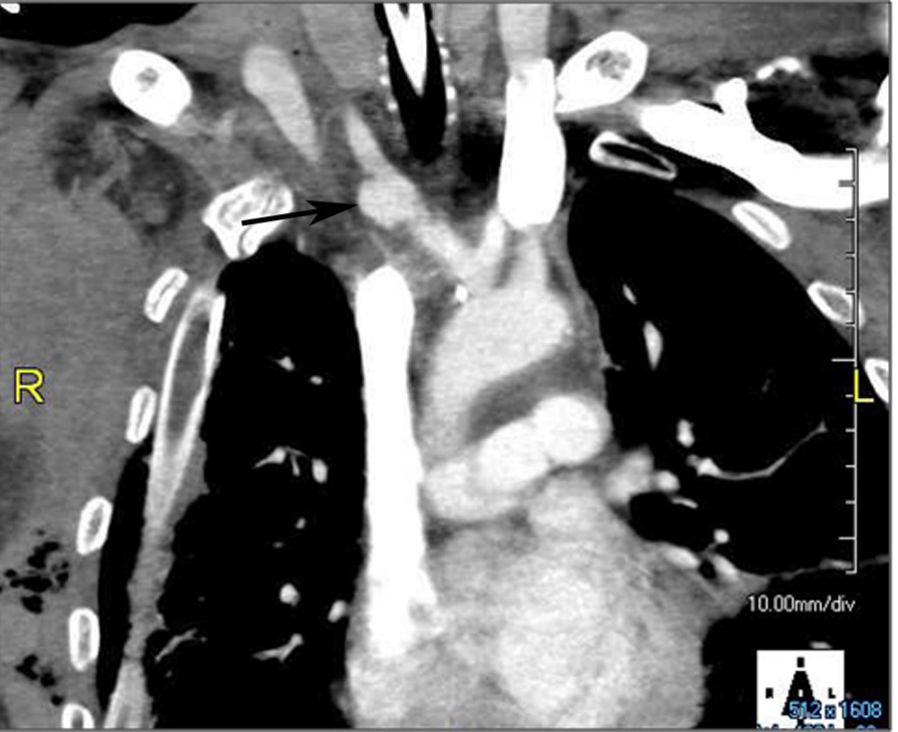
**Reconstructed computed tomography with contrast demonstrating a pseudoaneurysm at the junction of the right subclavian and common carotid arteries (arrow)**.

This is the second most commonly injured great vessel, with the proximal descending aorta the most common. Most innominate artery injuries occur at the vessel origin [7,19,20,28]. These are surgically repaired by placing a graft end-to-side from the ascending aorta and end-to-end to the distal innominate. Only after the graft is in place is the proximal innominate artery closed with pledgeted polypropylene sutures. An interposition graft or stent placement may be employed if the injury is in the mid portion of the vessel. More distal injuries (Figure 2) may require more complex reconstruction [20]. Generally all these injuries can be repaired without cardiopulmonary bypass or shunts although some authors recommend monitoring stump pressure [19].

#### Left carotid artery

**Figure 3:**
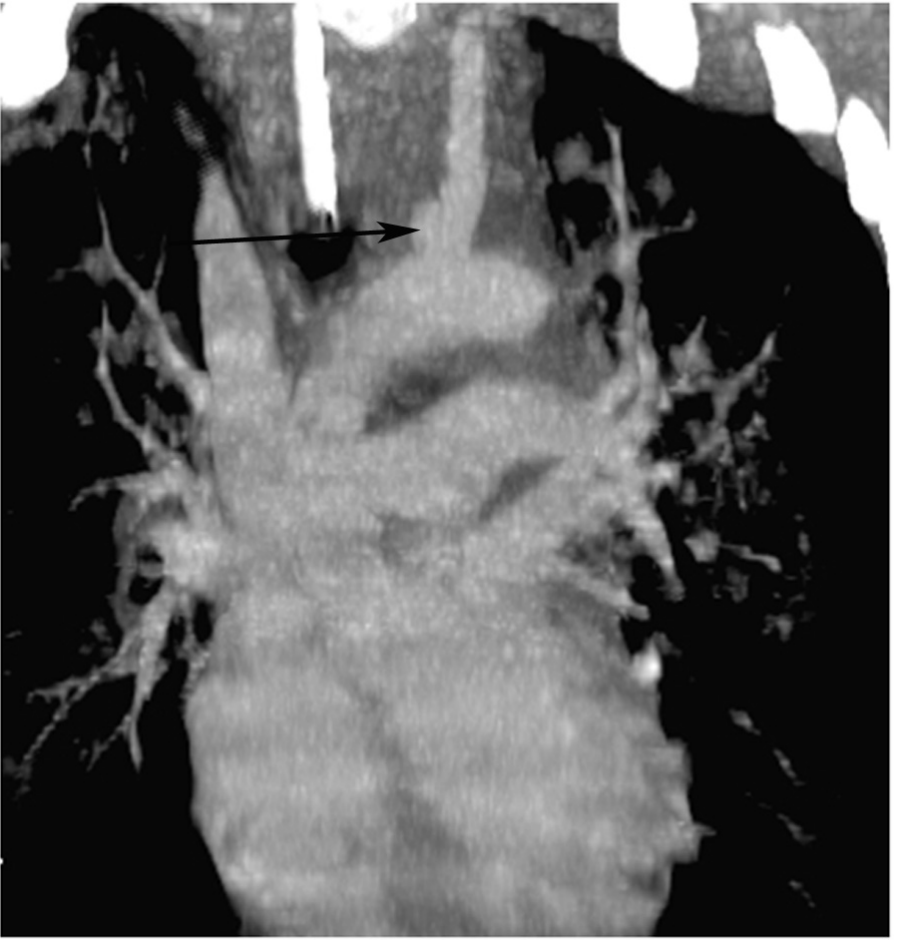
**Reconstructed computed tomography with contrast demonstrating a pseudoaneurysm at the origin of the left common carotid artery (arrow)**.

**Figure 4:**
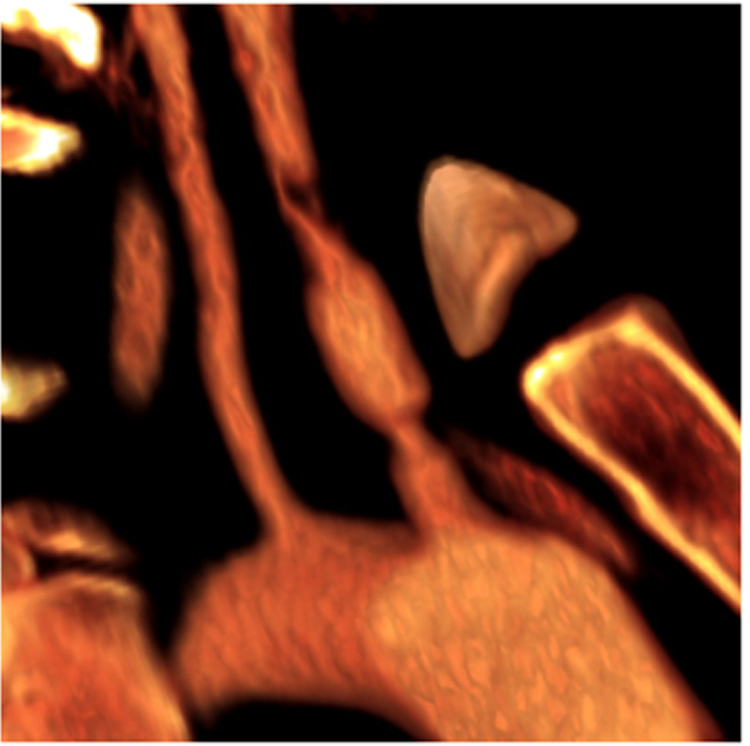
**"Three dimensional" rendering of injury depicted in Figure 3**.

**Figure 5:**
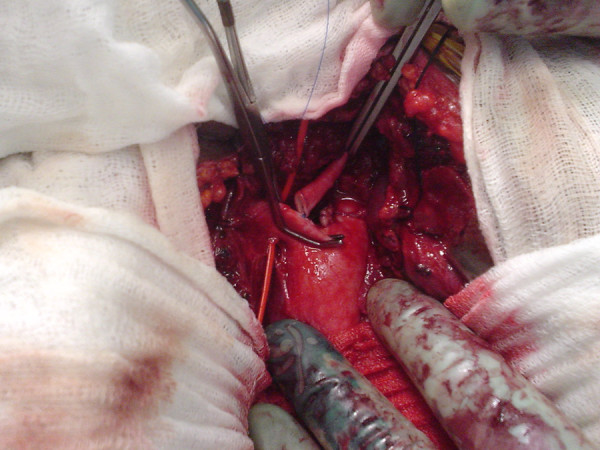
**Intra-operative photograph of and end-to side anastomosis of the left common carotid to the innominate artery**.

Similar to innominate injuries, left carotid injuries almost always occur at the origin[19,20,28]. (Figure 3, 4) Graft interposition or, less frequently, primary repair are used to repair these injuries. (Figure 5) As with innominate artery injuries, bypass and arterial shunts are rarely necessary.

#### Subclavian artery

**Figure 6:**
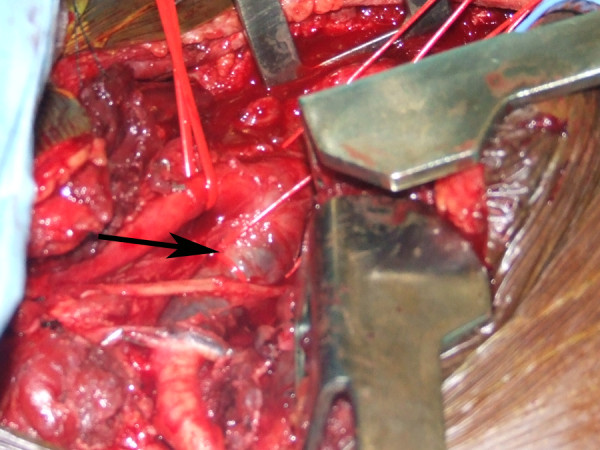
**Intra-operative photograph of a thrombosed right subclavian artery (arrow)**.

**Figure 7:**
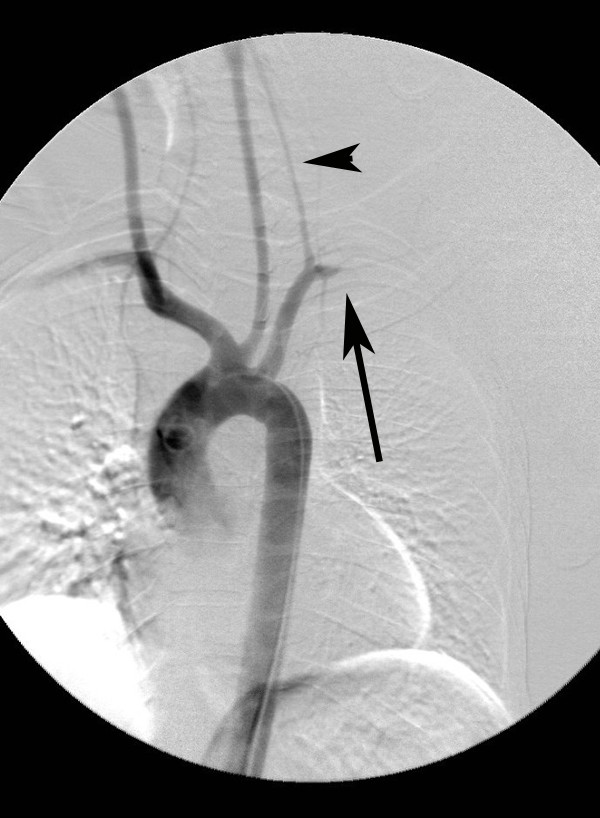
**Arch aortogram depicting thrombosed left subclavian artery (arrow) distal to the left vertebral artery (arrowhead)**.

Injury to this vessel often necessitates an interposition graft. Sternotomy with clavicular extension may be required to obtain optimal exposure of this difficult area, as blunt subclavian injuries tend to be more distal [8,12]. (Figure 6, 7) While interposition grafting is the most common method of reconstruction, primary repair may be feasible. Arterial ligation is uncommonly performed but may be life saving. Abundant collaterals prevent acute limb ischemia and, if it were to develop, re-vascularization can be performed. Interestingly, some authors have advocated not acutely re-establishing flow if the limb is not threatened and there is concomitant severe brachial plexus injury[8,13,55].

#### Pulmonary artery and vein

These are very rare injuries and the overwhelming majority of the injured die prior to hospitalization. The mortality of those who are alive on hospital admission is prohibitive[2,3].

#### Venous injuries

Thoracic caval injuries are exceedingly rare and highly lethal, especially if there is disruption of the atrio-caval junction. While superior vena caval injuries can rarely be repaired, injuries to the shorter intrathoracic inferior vena cava are almost uniformly fatal. Isolated azygous injury is exceedingly rare, limited to case reports and carries a significant mortality[56].

### Special Circumstances

Isolated venous injuries are uncommon but may carry a higher mortality than those to arteries[57]. More commonly they are associated with arterial trauma and, in some series, combined injuries are more lethal [16]. Another combination is trauma to the tracheo-bronchial tree with a great vessel injury[20,58-60]. These injuries require immediate operation with meticulous attention to airway management. Another unusual situation is blunt innominate injury in the setting where the left common carotid artery originates off of, or shares a common origin with, the innominate artery. While this anatomic variant is relatively common, it may complicate treatment. Several reports have described the successful management of this condition[61,62].

Excluding aortic trauma, blunt injury to the thoracic great vessels is infrequent and presents several challenges to the treating physicians and surgeons. On admission most patients are hemodynamically stable, have extra-thoracic injuries, may or may not have signs of limb ischemia, and often have a brachial plexus injury. An abnormal chest radiograph, especially a widened mediastinum, should prompt further imaging to precisely define the location and extent of the vascular injury. Unstable patients require immediate operation. Among stable patients, treatment options include operative management and endovascular intervention. This decision depends on the specific anatomy and availability of specialized personnel. Although these injures are associated with significant mortality and morbidity, rapid diagnosis and prompt intervention can yield gratifying results.

### Thoracic Aorta

Open repair requires single lung ventilation and a thoracotomy at the left fourth intercostal space. Today the aorta is rarely repaired with a clamp and sew technique due to the risk of paraplegia. Generally, the distal circulation beyond the proximal aortic clamp is perfused with oxygenated blood from an extracorporeal circuit. The circuit can lack an oxygenator and thus draw its blood from the left atrium via the inferior pulmonary vein or left atrial appendage. Our group has popularized use of femoral venous to femoral arterial cardiopulmonary bypass (CPB) as an alternative[63]. The advantage to the full CPB is the use of an integral pump sucker to rapidly deal with unexpected and life threatening bleeding encountered during the operation. Usually an interpostition graft is necessary as the aorta recoil results in defects of 2-3 inches, and surgeons strive to reduce tension on suture lines. However primary repair is prudent in certain cases. Despite major advances in surgical technique and adjunctive protective measures including spinal drainage, and distal aortic perfusion, open repair has significant morbidity and mortality. Rates of 18-28% operative mortality have been reported with paraplegia occurring in 2.3-14% of cases[64,65]. To date, our use of full CPB has not been associated with paraplegia. The associated injuries often seen with blunt aortic injury often preclude the necessary measures for open repair. Hypotension and anticoagulation in the setting of closed head injury is ill advised. Similarly, single lung ventilation can lead to hypoxia in the patient with pulmonary contusions.

**Figure 8:**
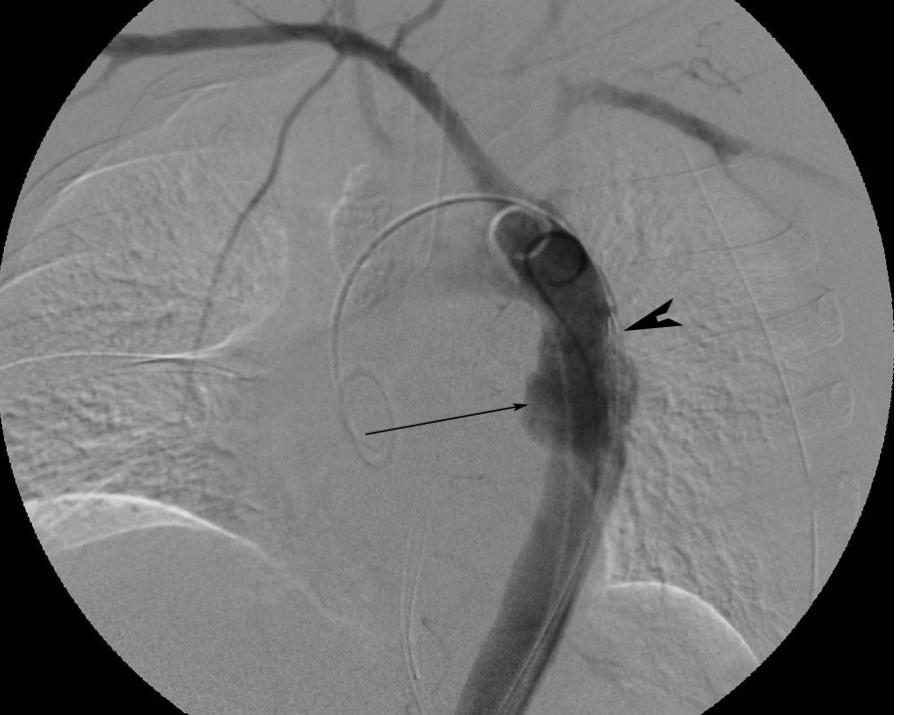
**Aortogram depicting aortic injury (arrow) with undeployed endovascular graft in position (arrowhead)**.

**Figure 9:**
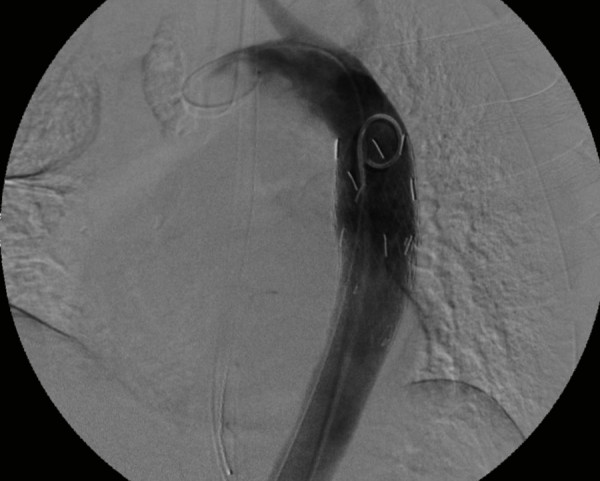
**Aortogram following endograft deployment with successful exclusion of the pseudoaneurysm**.

Endovascular stent grafts were initially described for treatment of abdominal aortic aneurysms by Parodi in 1991 [66]. The first reported case of endovascular stent graft repair of the thoracic aorta was reported by Dake and colleagues in for a patient with an enlarging pseudoaneurysm of the descending thoracic aorta[67]. Subsequent reports show successful placement and favorable outcomes for endovascular repair of aneurysms, traumatic injury and dissection[68]. Endovascular stent grafts are usually placed via a femoral artery cutdown. Iliac artery injury is a known complication, especially when these vessels are small [69]. A guide wire is placed under fluoroscopic guidance across the injury and the stent graft is deployed after angiography confirms the location of the injured segment. (Figures 8 and 9) The stent graft is a combination of metal stents providing radial force outwards with covered graft material that excludes flow from the injury. The advantages to endovascular stent grafting include minimal physiologic insult with access and deployment. There is no need for lateral decubitus positioning as in open thoracotomy which is advantageous in the head injured patient or pelvic fracture requiring external fixation. Finally, the ability to deploy the stent graft with no thorocotomy, aortic clamping, single lung ventilation, nor heparinization allows treatment of even the most critically injured or frail patients.

The American Association for the Surgery of Trauma (AAST) prospectively studied the treatment of blunt thoracic aortic injury at multiple institutions (AAST2) [70]. There have been significant changes since the landmark prospective AAST study from 1999 (AAST1) [22]. In 1999 there were no patients in the study treated with endografting, whereas 64.8% of patients underwent endografting in the 2008 study. Excluding patients in extremis, mortality decreased significantly form 22% to 13% and procedure-related paraplegia decreased from 8.7 to 1.6%. Of those patients who underwent endografting, the mortality was 7.2% (23.5% open mortality) and procedure related paraplegia was 0.8% (2.9% open paraplegia rate). However, the improvements in mortality and morbidity came at a price of 20% device related complication rate. The endoleak rate was 14.4% (18 patients) of which 6 underwent open repair.

At our institution endograft repair has become the primary treatment option for blunt aortic injury[71]. In our first 45 patients the mortality was 11% (none device related). There were no cases of paraplegia. However, similar to AAST2, the endoleak rate was 13.3% (6 patients) of which 3 patients had to undergo open repair.

It is clear that currently available devices were not designed for the small, sharply angulated aortic arches of young patients. The three thoracic endografts currently available in the US were designed for and approved by the FDA for nonruptured thoracic aortic aneurysms. The first available graft was the Gore TAG device. The Medtronic Talent device and the Cook Zenith TX2 device were later approved with an additional indication for penetrating aortic ulcers. There a variety of pitfalls that can lead to device failure, particularly in the treatment of blunt thoracic traumatic injuries. Over sizing of the stent or placement along the arch increases the risk of graft collapse[72,73]. To prevent graft failure, small diameter, short abdominal aortic cuffs from abdominal systems have been successfully used for these injuries [74-76]. The use of abdominal cuffs has several disadvantages: these cuffs tend to be short and require several grafts overlapping to cover the appropriate length. Short cuffs tend to be inflexible and are not well suited for conforming to the curve of the distal arch.

Devices that address these technical pitfalls are clearly needed. Home-made fenestrated devices designed to extend the area of coverage while maintaining patency of arch vessels have been used with success[77]. Multi-institutional trials designed to evaluate more flexible grafts are scheduled to start in the United States soon.

Despite their limitations, currently available endoluminal stent grafts have been used with promising results. A recent meta-analysis of seventeen retrospective cohort studies, demonstrated a significantly lower procedure related mortality, overall 30 day mortality and postoperative paraplegia in patients treated with endografts vs. open repair[78,79].

Fortunately, in our experience, there have been no midterm graft failures or need for intervention. However, the durability and long term outcomes for endovascular stent grafts are unknown in these typically young patients. Long term follow-up will be required. Follow-up can be difficult in this group of patients. Additionally the use of radiologic imaging over a long period of time carries with it a tangible risk of future malignancy [80].

The treatment of blunt aortic injury has undergone a radical paradigm shift with the introduction of endovascular stent grafts. With the evolution of graft design and successive models conforming to the curve of the aortic arch and produced in smaller diameter sizes, it is likely that endovascular repair will become the primary treatment in the majority of blunt aortic injury with improved morbidity and mortality rates for these often challenging injuries.

## Competing interests

The authors declare that they have no competing interests.

## Authors' contributions

JO contributed directly to drafting of the manuscript. CB contributed directly to drafting of the manuscript. TS contributed directly to drafting of the manuscript and participated in its organization. BG contributed directly to drafting of the manuscript. DN contributed directly to drafting of the manuscript, conceived the work, and coordinated its design. All authors read and approved the final manuscript.
